# The association of FRAX with predictors of falls in the assessment of postmenopausal osteoporosis in Turkey: the fracture study of Turkey (FRACT study)

**DOI:** 10.1007/s11657-024-01387-2

**Published:** 2024-04-20

**Authors:** Sansin Tuzun, Ulku Akarirmak, Bilal Kulaksiz, Aslinur Keles, Dilara Okutan, Pinar Karsli, Sevgi Selin Kurtoglu, Eren Aygun, Deniz Palamar

**Affiliations:** Department of Physical Medicine and Rehabilitation, Istanbul University-Cerrahpasa, Cerrahpasa Medical Faculty, Fatih, Istanbul, Turkey

**Keywords:** FRAX, Fall risk predictors, Balance, Postmenopausal osteoporosis

## Abstract

**Summary:**

Although FRAX is used for fracture risk evaluation, this tool does not include balance and fall risk. The association between the predictors of falls and high FRAX scores we found in this study suggests that risk indicators for falls may add substantial value to FRAX by improving fracture risk prediction.

**Purpose:**

This observational, descriptive, and cross-sectional study aimed to assess the fall risk predictors and explore their association with FRAX in Turkish patients with postmenopausal osteoporosis.

**Methods:**

Two hundred and nine (209) women with postmenopausal osteoporosis referred to the Fracture Liaison Service (FLS) at Istanbul University–Cerrahpaşa were enrolled in the FRACT study (The Fracture Study of Turkey). Clinical risk factors were assessed using the FRAX tool. Tandem stance, Tandem walking, Timed up and go (TUG), and Chair stand tests were performed to assess balance and fall risk.

**Results:**

Among patients with a mean age of 67.6 (± 9.7) years, 66 patients (31.6%) had osteoporosis without fractures and 143 patients (68.4%) had fragility fractures. The proportion of patients with poor performance of fall prediction tests was significantly higher in patients with a fragility fracture than those with osteoporosis alone. There was an inverse relationship between dynamic balance tests and the reported number of prior falls in the past year. FRAX score was higher in patients with impaired Tandem stance, Tandem walking, and TUG tests (*p* = 0.008, *p* = 0.035, *p* = 0.001, respectively).

**Conclusion:**

Assessment of fall risk predictors should be one of the major pillars in the physical evaluation of osteoporotic patients in the FLS setting. FRAX is a useful tool to determine the fracture risk of patients with both static and dynamic balance impairments. Combining balance assessment with FRAX may be an important step to optimize osteoporosis risk assessment.

## Introduction

Osteoporosis and related fractures pose a growing burden on patients, healthcare providers, and the economy [1]. Globally, the number of osteoporotic fractures is expected to double between 2010 and 2040 [2]. These fractures can lead to functional disability, reduced quality of life, and healthcare resource utilization [3]. Patients who had previous fractures often suffer from impaired postural stability, which can result in an increased risk of falls and further fractures [4, 5].

Multiple risk factors, aside from bone mineral density (BMD), contribute to fracture risk [6]. Balance impairment and falls are common among older adults, especially those with osteoporosis. Fall-related risk factors are the most common cause of fractures and often overlap with risk factors for osteoporosis, highlighting the need for integrated fall and fracture services [7]. Reducing the incidence of falls is a primary prevention strategy that can improve quality of life and reduce healthcare costs [8]. To that end, the Centers for Disease Control and Prevention (CDC) recommends screening for falls at every visit for patients with osteoporosis [9]. Balance impairment because of aging, comorbidities, or medication may show different patterns according to populations living in different countries and have to be assessed. Preventing falls should also prevent future fractures, though the empirical evidence for this is poor [10].

FRAX® is the most used tool for assessing the risk of fractures, which combines clinical risk factors and BMD at the femoral neck to calculate fracture probability at an individual patient level [11]. The use of country-specific FRAX models is recommended in recent osteoporosis treatment guidelines to evaluate the likelihood of fractures in postmenopausal women [12]. Although FRAX models are widely used for fracture risk evaluation, they do not consider balance and fall risk. Indeed, studies with regard to the relationship between FRAX and predictors of falls such as balance tests are quite limited [13, 14]. This study aimed to assess the fall risk predictors and to explore their association with FRAX in a population consisting of Turkish patients with postmenopausal osteoporosis.

## Materials and methods

### Study design

The FRACT study (The Fracture Study of Turkey), conducted at the Department of Physical Medicine and Rehabilitation at Istanbul University–Cerrahpasa, was an observational and descriptive registry study designed to collect follow-up data from postmenopausal women with osteoporosis in Turkey. Patients with fragility fractures were registered for post-fracture care and received close follow-up at FRACT, the first FLS in Turkey, in line with the European Guidance on Postmenopausal Osteoporosis [12]. The study followed the guidelines of the 1964 Helsinki Declaration and its subsequent amendments. The study was approved by the Medical Ethics Committee of the university (IUC-83045809–604.01.02), and all participants provided written informed consent before enrolling. Although the sample size of the study was planned as 600 patients using the G-Power program, due to the COVID-19 pandemic, it was decreased to 200 with an amendment approved by the Medical Ethics Committee.

### Study population

Female patients aged 50 or older who were diagnosed with osteoporosis based on BMD according to the WHO criteria [15], or who had a prior fragility fracture caused by low-energy trauma regardless of their BMD values, were screened. Between March 2018 and July 2020, clinicians referred patients to the FLS, from which 218 patients diagnosed with postmenopausal osteoporosis were enrolled in the study. Nine patients were excluded because of a history of metastatic cancer and neurological and orthopedic disorders that may hinder functional assessment. Two hundred and nine women with postmenopausal osteoporosis were evaluated in this study.

The participants were divided into two groups: Group 1 included patients with postmenopausal osteoporosis without fractures and *T*-scores <  − 2.5, while Group 2 consisted of patients with fragility fractures regardless of their BMD.

### Data collection

All evaluations were conducted by experienced physiatrists in Physical Medicine and Rehabilitation Department of İstanbul University-Cerrahpaşa, who collected information on demographic data, including age, sex, gynecologic profile (age at menopause, number of delivery, duration of lactation (months)), and comorbidities such as diabetes mellitus, hyperthyroidism, hypogonadism, premature menopause, chronic malnutrition, malabsorption, chronic liver disease, and rheumatologic diseases. Clinical risk factors for osteoporotic fractures were assessed using the FRAX tool for Turkey, which takes into account age, sex, weight, height, BMI, previous fracture, hip fracture of parents, current smoking, glucocorticoid usage, rheumatoid arthritis, alcohol consumption, and secondary osteoporosis. FRAX probabilities of a major osteoporotic fracture (MOF) were categorized as above or below the age-dependent intervention thresholds recommended for Turkey [16] and thereafter categorized as “high” or “low” FRAX score. A physical examination was conducted to assess thoracic hyperkyphosis and fall risk, which included an inquiry about the number of falls (0, 1–2, and 3 or more) in the previous year, as well as a clinical evaluation of balance with the Tandem stance, Tandem walking, Timed up and go (TUG), and Chair stand tests, which have been well established, also in our country.

The Tandem stance balance test was used to assess static balance, and patients were asked to place one foot in front of the other, heel touching toes, and hold this position for 10 s without moving their feet or needing support [17]. Tandem walking involved asking patients to walk in a straight line with one foot immediately in front of the other (heel to toe), with arms down by their side. Patients who were able to perform eight steps in a smooth, continuous rhythm were considered to have a normal gait [18]. For the Chair stand test, patients were asked to stand up and sit down from a chair without armrests five times as fast as possible, with their arms crossed over their chests. The TUG test was used to assess dynamic balance and functional mobility and involved asking patients to rise from a standard armchair, walk to a marker 3 m away, turn, walk back, and sit down again [19, 20]. Patients who took 12 s or more to complete the TUG test were considered to be at an increased risk for falling [21].

Routine laboratory investigations were performed, including complete blood cell count, erythrocyte sedimentation rate (ESR), alanine aminotransferase (ALT) and aspartate aminotransferase (AST) levels, creatinine and blood urea levels, electrolytes, calcium (Ca) and phosphate (P) levels, thyroid function tests (TFTs), alkaline phosphatase, 25(OH) vitamin D, and parathyroid hormone (PTH). X-rays of the dorsal and lumbar regions were conducted to assess vertebral heights, and BMD evaluations were performed using dual X-ray absorptiometry (DXA) scan referencing to NHANES III values, at the Nuclear Medicine Department with a Hologic QDR 4500SL (S/N 45624) (Bedford, MA). Osteoporosis was defined as a *T*-score of − 2.5 at the lumbar spine, femur neck, or total hip. The fracture risk assessed with the FRAX tool was used to calculate the 10-year major osteoporotic and hip fracture risks of the patients based on the BMD (g/cm^2^) of the femoral neck [12].

### Statistical analysis

The descriptive statistics of continuous data were presented as mean and standard deviation with the latter expressed in parentheses next to the mean. Categorical data were presented as percentages. To test the differences between the two groups with continuous data, a “bootstrapped independent samples *t*-test” was used, and the equality of variances was checked. The analysis involved 1000 bootstrap replicates, which is a common practice. Chi-square or Fisher’s Exact test was used to test the significance of contingency tables, depending on the appropriate criteria. To evaluate the correlation between a nominal variable and an ordinal one in a contingency table, Cramer’s V was calculated. A *p* value less than or equal to 0.05 was considered statistically significant. All statistical analyses were performed using R Program 4.1.0.

## Results

Sixty-six patients (31.6%) had osteoporosis without fractures (Group 1) and 143 patients (68.4%) had prior fragility fractures regardless of their BMD (Group 2). The fractures were located at various sites, including the vertebrae (*n* = 65), wrist (*n* = 37), hip (*n* = 17), lower leg (*n* = 20), arm (*n* = 17), and clavicle (*n* = 1). Thirty-one patients (14.7%) had secondary osteoporosis due to various conditions such as rheumatoid arthritis, type 1 diabetes mellitus, hyperthyroidism, chronic liver disease, glucocorticoid usage, and aromatase inhibitor usage for breast cancer.

The average age of our patients was 67.6 (± 9.7) and mean BMI was 27.6 (± 5.2). Thoracic hyperkyphosis was observed in 79% of patients with a prior fracture, compared to 50% of patients without fractures (*p* = 0.001). Although the mean calcium level was within the normal range in both groups, there was a significant difference between the fractured and non-fractured groups (*p* = 0.034). The average vitamin D level was 25.8 ng/ml in the fractured group, which was significantly lower than the non-fractured group (*p* = 0.021). The demographic and detailed baseline characteristics of the postmenopausal women are provided in Table 1. At our FLS center, patients with postmenopausal osteoporosis are routinely screened with a questionnaire that also includes gynecologic profile (such as age at menopause, number of delivery, duration of lactation). Additionally detailed laboratory measurements are taken to guide optimal management strategies. Neither the variables of the gynecologic profile nor the laboratory measurements not shown in the table were statistically significant.

**Table 1:** Demographic features and baseline characteristics of postmenopausal women with osteoporosis (*n* = 209)

Variable	Overall	Group 1 (non-fractured)	Group 2 (fractured)	*p* value
*n*	209	66	143	
Age (years)	67.6 ± 9.7	64.8 ± 8.6	69.0 ± 9.9	*0.001*
BMI (kg/m^2^)	27.6 ± 5.2	27.0 ± 5.5	27.9 ± 5.1	0.254
Thoracic hyperkyphosis				
No Yes	63 (30.1) 146 (69.9)	33 (50.0) 33 (50.0)	30 (21.0) 113 (79.0)	*0.001*
Regular exercise*				
No Yes	120 (57.3) 89 (42.7)	38 (57.6) 28 (42.4)	82 (57.1) 61 (42.9)	0.951
Self-reported falls in the previous year				
0 falls 1–2 falls ≥ 3 falls	133 (63.8) 64 (30.6) 12 (5.6)	44 (66.7) 18 (27.8) 4 (5.5)	89 (62.3) 46 (32.1) 8 (5.6)	0.850
Laboratory				
Calcium (mg/dl)	9.4 ± 0.7	9.6 ± 0.5	9.4 ± 0.8	*0.034*
Phosphate (mg/dl)	3.5 ± 0.6	3.4 ± 0.6	3.5 ± 0.6	0.190
ALP (U/L)	68.4 ± 24.4	64.5 ± 21.4	70.0 ± 25.4	0.136
25(OH)D (ng/ml)	27.7 ± 14.4	32.1 ± 17.6	25.8 ± 12.2	*0.021*
PTH (pg/ml)	58.0 ± 35.4	52.3 ± 24.6	60.2 ± 39.3	0.119
Albumin (gr/dl)	4.3 ± 0.6	4.4 ± 0.5	4.2 ± 0.6	0.364
FRAX (%)				
FRAX Probability of HF	4.6 ± 5.2	2.8 ± 2.7	5.5 ± 5.8	*0.001*
FRAX Probability of MOF	12.7 ± 8.0	8.3 ± 4.9	14.6 ± 8.3	*0.001*
DXA (*T*-score)				
Lumbar spine (L1–L4)	− 2.17 ± 1.16	− 2.39 ± 1.08	− 2.08 ± 1.18	0.072
Femoral neck	− 1.99 ± 0.87	− 2.03 ± 0.81	− 1.99 ± 0.89	0.771

Italics represent significant differences between groups. Data presented as mean ± SD or *n* (%)

*BMI* body mass index, *ALP* alkaline phosphatase, *25(OH)D* 25-hydroxy vitamin D, *PTH* parathyroid hormone, *SGOT* serum glutamic oxaloacetic transaminase, *SGPT* serum glutamic pyruvic transaminase, *TSH* thyroid-stimulating hormone, *HF* hip fracture, *MOF* major osteoporotic fracture

^*^Regular exercise is defined as approximately 150 min of moderate-intensity exercise per week

The distribution of patients based on the Turkish FRAX intervention threshold was as follows: 59 (34.3%) patients were below the intervention cut-off value, while 113 (65.7%) patients were above the cut-off value. Among the patients who had experienced a prior fracture, 77% had a FRAX MOF-score equal to or above the intervention threshold. In contrast, only 39.6% of patients without fractures had a FRAX score equal to or above the threshold. As expected, FRAX scores were significantly higher in patients with postmenopausal osteoporosis who had a fragility fracture (*p* < 0.001) (see Table 1).

**Fig. 1:**
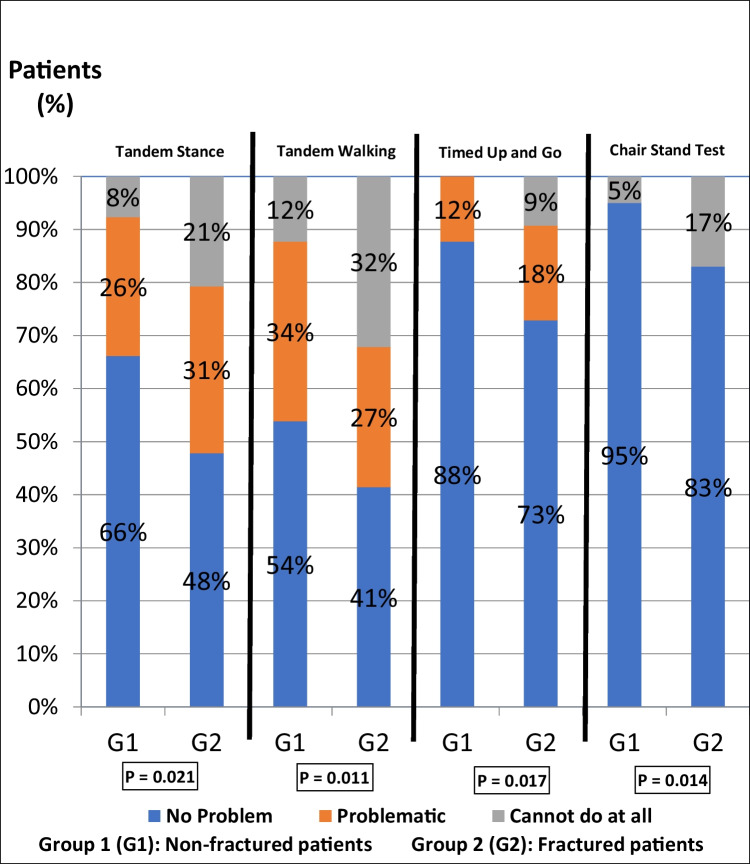
Fall risk predictors in the fractured (Group 1) and non-fractured (Group 2) patients with postmenopausal osteoporosis. *p* < 0.05 was considered statistically significant and presented in bold. The proportion of patients with poor performance of fall prediction tests was significantly higher in patients with a fragility fracture than those with osteoporosis alone

As shown in Fig. 1, more patients with past fractures had difficulties in completing the fall risk prediction tests than those with no prevalent fracture. These differences were statistically significant in Tandem stance, Tandem walking, TUG performance, and Chair stand tests (*p* = 0.021, *p* = 0.011, *p* = 0.017, *p* = 0.014, respectively).

Baseline differences of the patients in terms of FRAX scores are presented in Table 2. There was no significant difference with regard to the number of self-reported falls in the high and low FRAX score groups. However, age (*p* = 0.001) and *T*-score at the femur neck (*p* = 0.013) were significantly different.

**Table 2:** The baseline differences between those below (low FRAX score) and equal and above the treatment threshold (high FRAX score) according to Turkey-specific FRAX (*n* = 172)

Variable	Overall	Low FRAX score	High FRAX score	*p* value
*n*	172	59	113	
Age (years)	67.4 ± 9.9	60.9 ± 6.4	70.8 ± 9.8	*0.001*
BMI (kg/m^2^)	27.4 ± 5.1	27.6 ± 5.3	27.3 ± 5.0	0.709
DXA (*T*-score)				
Lumbar spine (L1–L4)	− 2.16 ± 1.13	− 2.09 ± 1.14	− 2.19 ± 1.13	0.580
Femoral neck	− 1.97 ± 0.86	− 1.73 ± 0.86	− 2.10 ± 0.83	*0.013*
Self-reported falls in the previous year 0 falls				
1–2 falls ≥ 3 falls	108 (62.8) 54 (31.4) 10 (5.8)	39 (66.1) 17 (28.8) 3 (5.1)	69 (61.1) 37 (32.7) 7 (6.2)	0.850

Italics represent significant differences between groups. Data presented as mean ± SD or *n* (%)

*BMI* body mass index

**Fig. 2:**
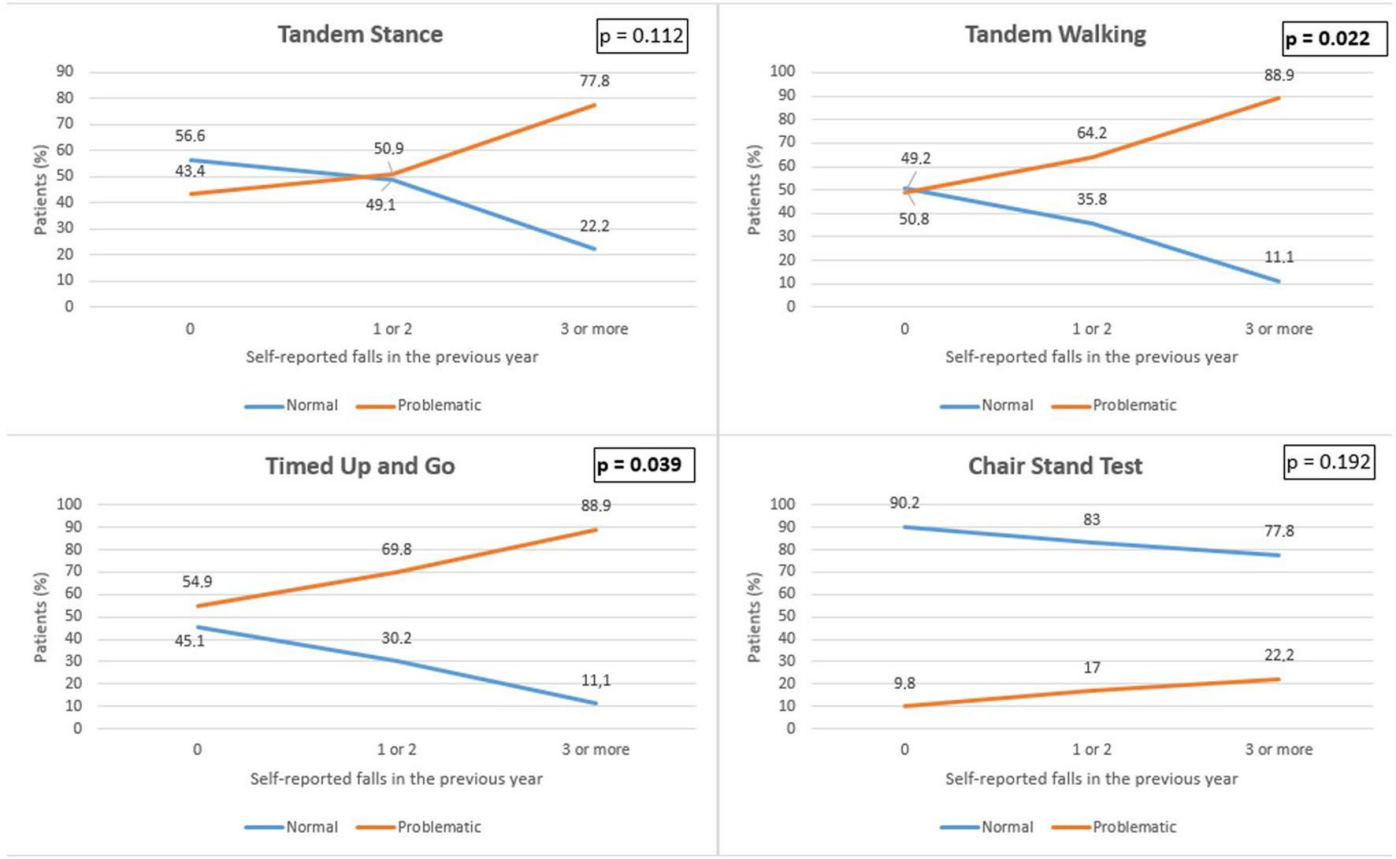
The association between the number of falls and fall risk predictors. *p* < 0.05 was considered statistically significant and presented in bold. There was a moderate correlation between Tandem walking and the frequency of falls (*V* = 0.204, *p* = 0.022), and a weak correlation between the TUG performance test and the frequency of falls (*V* = 0.189, *p* = 0.034)

Among the patients, 76 (36.2%) had experienced at least one fall, while 12 (5.6%) had encountered 3 or more falls in the previous year. An inverse relationship was found between dynamic balance tests and the number of falls. The proportion of the patients who successfully completed the Tandem walking test was 50.8% in those without a history of falls, 35.8% in those with a history of 1 or 2 falls, and 11.1% in those with a history of 3 or more falls (*p* = 0.022). For the Tandem Stance test the comparative rates were 56.6%, 49.1%, and 22.2%, respectively (*p* = 0.112). In total, 45.1% of patients with no falls history satisfactorily completed the TUG test whereas 30.2% of patients did so with a history of 1 or 2 falls, and 11.1% with 3 or more falls (*p* = 0.039). On the other hand, the proportion of patients who could perform the Chair stand test was 90.2%, 83%, and 77.8%, respectively (*p* = 0.192). There was a moderate correlation between Tandem walking and the frequency of falls (*V* = 0.204, *p* = 0.022), and a weak correlation between the TUG performance test and the frequency of falls (*V* = 0.189, *p* = 0.034). The association between the number of falls and fall risk predictors is illustrated in Fig. 2.

**Fig. 3:**
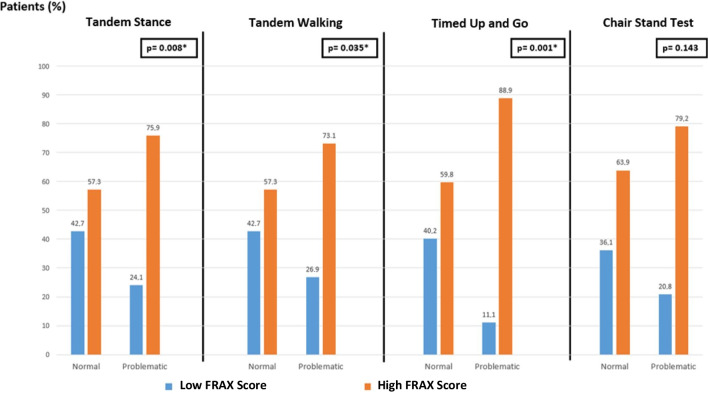
The association between FRAX scores and fall risk predictors. *p* < 0.05 was considered statistically significant and presented in bold. The proportion of patients with high FRAX scores was significantly higher in those with poor performance in Tandem stance, Tandem walking, and TUG tests

There was a relationship between FRAX and clinical predictors of falls. Balance tests such as Tandem stance, Tandem walking, and TUG tests are highly related to fall risk, and we found that the FRAX score was higher among patients with impaired balance tests. In total, 75.9% of the patients who could not perform the Tandem stance test had high FRAX scores, whereas only 57.3% of the patients who satisfactorily completed the test had high FRAX scores (*p* = 0.008). For the Tandem walking test, the comparable rates were 73.1% and 57.3%, respectively (*p* = 0.035). In total, 88.9% of patients with poor TUG performance had high FRAX scores compared to 59.8% in those with completed TUG performance (*p* = 0.001). Chair stand was the only test that did not reach statistical significance (*p* = 0.143). Results related to FRAX score and clinical predictors of falls are displayed in Fig. 3.

## Discussion

This study has shown significant associations between tests predictive of falls with a past history of falls, a prior fracture, and FRAX scores above the Turkish intervention threshold. These findings imply a relation between FRAX and fall risk in Turkey.

Higher fracture risk is related to a greater propensity to fall. Impaired balance is the leading risk factor for falls in the elderly, and there is a strong correlation between balance deficit and the incidence of falls [22]. Osteoporotic postural changes like hyperkyphosis, as seen in many of our patients with a prior fracture (79%), can increase the risk of falls leading to a forward-stooping posture, and this causes a vicious circle between falls and fractures in postmenopausal women [23]. Indeed, the proportion of patients with poor performance on all fall prediction tests was significantly higher in patients with a fragility fracture than those with osteoporosis alone in our study. These findings are in line with previous studies by Wang et al. and Bhattacharya et al., who found that patients with osteoporotic fractures had poorer static and dynamic balance performance than control patients without osteoporosis [24, 25].

There was a strong relationship between self-reported fall history and some of the predictors of falls. In particular, Tandem walking and TUG performance were negatively correlated with the number of falls in the previous year, revealing that as balance tests deteriorate, the number of falls increases. It reveals that among fall risk predictors, dynamic balance tests such as Tandem walking and TUG, which involve walking, are more relevant to the history of falls.

In an earlier study, high FRAX scores were associated with poor outcomes in some balance tests [13]. Although The FRAX tool does not include a direct assessment of fall risk, it has been shown that the risk factors included in the tool do capture some elements of the fracture risk associated with falls [14]. This observation is consistent with our finding that patients with high FRAX scores had a higher rate of impaired fall prediction tests (TUG performance, Tandem stance, and Tandem walking tests). Among them, the TUG performance test showed the strongest association with FRAX. This test is commonly used to evaluate balance and fall risk in the elderly [26] and proved to be efficient for assessment in Turkish postmenopausal osteoporotic patients as well. A systematic review has shown that the TUG test is linked to a history of past falls; however, its ability to predict future falls may be more limited [27]. The Chair stand test was the only fall prediction parameter that was not significant in patients with a high FRAX score. In fact, this test is more an indicator of lower extremity strength than of balance [28].

Taking into account that our study group consists of postmenopausal women, current literature on the association between muscle performance and fracture risk in women is controversial whereas this relationship is more evident in men [29]. This highlights the advantage of using dynamic balance tests in clinical practice in the FLS setting. Another point that should be emphasized is that most studies examining fall risk predictors were performed in geriatric or community-dwelling populations [30]; our study, however, was conducted in postmenopausal women who are relatively younger, reflecting real-world evidence at a FLS center in our country.

The association between the indices of fall risk and high FRAX scores suggests that FRAX captures some element of fall risk as documented recently [31]. The proportion captured is, however, small and a fall history identifies a substantial fracture risk over and above that provided by FRAX [31–33] This in turn suggests that risk indicators for falls may add substantial value to FRAX by improving fracture risk prediction. The suggestion is consistent with our findings but falls short of proof. This would demand a prospective study with fracture outcomes.

Fall intervention programs, including targeted exercises to increase physical fitness and balance, are crucial in community healthcare due to the high risk of falls and fractures among the elderly. However, the existing literature does not clearly indicate whether weight loss is the key to improving balance in fall intervention programs. While one study found no significant difference in BMI between fallers and non-fallers [34], another study linked obesity to balance disturbances [35]. Our study population was clinically overweight, with a mean BMI of 27.6. In countries like Turkey, where obesity is a prevalent healthcare issue [16], weight loss could still be a beneficial recommendation for improving balance.

Vitamin D may have positive effects on fall and fracture risk reduction [36, 37]. In the present study, the vitamin D level of the fractured group was lower than non-fractured patients. The mean vitamin D level of the fractured patients was below 30 ng/ml indicating insufficiency. This value has been also shown in one study as a cut-off value for maintaining muscle function and reducing the risk of falling [38]. However, there was no relationship between vitamin D level and fall history in our study. Prospective studies in larger series are needed to comment on this issue. Risk factors for falls should be identified with regard to the nation as well.

Although the mean calcium level was within the normal range in both groups, the calcium level of the fractured group was lower than that of non-fractured patients. Non-pharmacological management of osteoporosis is an important aspect; therefore, adequate intake of calcium, preferably achieved through dietary intake or calcium supplementation, could be a part of the prevention of fragility fractures [39]. Meta-analyses of combined calcium and vitamin D supplements have demonstrated a reduction in hip and non-vertebral fractures, as well as vertebral fractures [40]. Consequently, at every stage of life, adequate dietary intakes of calcium and vitamin D contribute to bone health, especially for those at risk of osteoporosis and/or fragility fractures.

A fall assessment should be undertaken in all patients with osteoporosis and fragility fractures; those at risk should be offered exercise programs to improve balance and/or that contain a combined exercise protocol [39]. It seems that multicomponent exercise therapies that integrate strength/resistance training with gait, balance, and functional training are more successful. The goal of encouraging frequent, supervised physical exercise is to improve balance and lower the chance of falling in the future. [41, 42]

Regrettably, the COVID-19 pandemic had a notable detrimental effect on the recruitment process of our study, leading to a smaller sample size than anticipated. Fewer patients were included in the study than the estimated number due to restrictions imposed on individuals over 65 years of age in Turkey who could not visit our outpatient clinic. However, the study included a high-risk population that was expected to benefit the most from anti-osteoporotic interventions and the FLS approach. There are a number of further limitations to consider. Of particular note is that the study was cross-sectional and no fracture outcomes were gathered prospectively. Also, it is uncertain whether the risk indicators for falls in the present study add information over that provided by a fall history.

In conclusion, the assessment of fall risk predictors should be one of the major pillars in the physical evaluation of osteoporotic patients in the FLS setting in Turkey. An accurate assessment of balance and fall risk is an essential aspect, not only in the geriatric population but also in all postmenopausal women with osteoporosis. This should encompass both static and dynamic balance tests such as Tandem stance, Tandem walking, and TUG performance with well-established associations to fall risk. FRAX seems a useful tool to determine fracture risk also in patients with both static and dynamic balance impairments.

## Data Availability

The data that support the findings of this study are available from the corresponding author, upon reasonable request.
